# Predominant Expression of Hybrid *N*-Glycans Has Distinct Cellular Roles Relative to Complex and Oligomannose *N*-Glycans

**DOI:** 10.3390/ijms17060925

**Published:** 2016-06-13

**Authors:** M. Kristen Hall, Douglas A. Weidner, Yong Zhu, Sahil Dayal, Austin A. Whitman, Ruth A. Schwalbe

**Affiliations:** 1 Department of Biochemistry and Molecular Biology, Brody School of Medicine, East Carolina University, 600 Moye Boulevard, Greenville, NC 27834, USA; hallma@ecu.edu (M.K.H.); dayals13@students.ecu.edu (S.D.); whitmana13@students.ecu.edu (A.A.W.); 2 Department of Microbiology and Immunology, Brody School of Medicine, East Carolina University, 600 Moye Boulevard, Greenville, NC 27834, USA; weidnerd@ecu.edu; 3 Department of Biology, East Carolina University, 1000 E. 5th Street, Greenville, NC 27858, USA; zhuy@ecu.edu

**Keywords:** glycobiology, *N*-glycan, transmembrane glycoprotein, cell surface glycan, cell–cell adhesion, cell motility, lateral heterogeneity of proteins in membranes, cadherin

## Abstract

Glycosylation modulates growth, maintenance, and stress signaling processes. Consequently, altered *N*-glycosylation is associated with reduced fitness and disease. Therefore, expanding our understanding of *N*-glycans in altering biological processes is of utmost interest. Herein, clustered regularly interspaced short palindromic repeats/caspase9 (CRISPR/Cas9) technology was employed to engineer a glycosylation mutant Chinese Hamster Ovary (CHO) cell line, K16, which expresses predominantly hybrid type *N*-glycans. This newly engineered cell line enabled us to compare *N*-glycan effects on cellular properties of hybrid type *N*-glycans, to the well-established Pro^−^5 and Lec1 cell lines, which express complex and oligomannose types of *N*-glycans, respectively. Lectin binding studies revealed the predominant *N*-glycan expressed in K16 is hybrid type. Cell dissociation and migration assays demonstrated the greatest strength of cell–cell adhesion and fastest migratory rates for oligomannose *N*-glycans, and these properties decreased as oligomannose type were converted to hybrid type, and further decreased upon conversion to complex type. Next, we examined the roles of three general types of *N*-glycans on ectopic expression of E-cadherin, a cell–cell adhesion protein. Microscopy revealed more functional E-cadherin at the cell–cell border when *N*-glycans were oligomannose and these levels decreased as the oligomannose *N*-glycans were processed to hybrid and then to complex. Thus, we provide evidence that all three general types of *N*-glycans impact plasma membrane architecture and cellular properties.

## 1. Introduction

*N*-glycosylation is one of the most vital, widespread, and complex co- and post-translational protein modifications [1]. All *N*-glycans share a common core sugar sequence and are classified as three major types: oligomannose, complex, and hybrid [2]. The actions of *N*-acetylglucosaminyltransferases (GlcNAcTs, coded by *Mgat* genes) are responsible for initiating the different branch points on the common pentasaccharide core which leads to the three major types of *N*-glycans [2]. The elongation of each branch point depends on the presence of numerous elongation enzymes and transporters, as well as substrate availability, and thus adds to the microheterogeneity of *N*-glycan structures [2]. GlcNAcT-I (coded by *Mgat1*) and GlcNAcT-II (coded by *Mgat2*) are responsible for converting oligomannose type to hybrid type, and hybrid type to complex type, respectively [2]. Knock out mice of *Mgat1* and *Mgat2* resulted in death around embryonic day 9 [3], and during early post-natal development [4], respectively. These studies revealed the importance of hybrid and more importantly complex type *N*-glycans for development of mammals, and furthermore that oligomannose type *N*-glycans are inadequate for embryonic development.

Carbohydrate-dependent interactions that modulate cell adhesion, motility, and signaling have been described as three distinct types of glycosynapses [5,6]. Here the type 3 glycosynapse is of interest since one of the components involves *N*-linked glycans of an integrin receptor [5,6]. *N*-linked glycans are attached to the extracellular regions of many transmembrane proteins and thereby contribute to the plasma membrane architecture and cellular properties. Changes in *N*-glycan structures at the cell surface alter the spatial arrangement of a major cell–cell adhesion molecule (E-cadherin) [7,8], and voltage gated potassium channels (Kv3.1b and a) [7,9,10] in the plasma membrane. Further these modifications in spatial arrangement correlated with changes in cell–cell adhesion and cell migration, respectively. E-cadherin is a major component of adherens junctions of epithelial cells [11]. In agreement with enhanced E-cadherin-dependent cell–cell adhesion by oligomannose type relative to complex type *N*-glycans in Chinese Hamster Ovary (CHO) cells which lack adherens junction [7], expression of oligomannose *N*-glycans strengthened adherens junctions as compared to complex *N*-glycans [12]. Increased levels of complex *N*-glycans with β1,6 *N*-acetylglucosamine (catalyzed by GlcNAcT-V) on E-cadherin were more recently shown to cause weakening of E-cadherin mediated cell–cell adhesion leading to increased tumor progression [13], and the same study demonstrated that modification of E-cadherin with bisected *N*-glycans (catalyzed by GlcNAcT-III) increases the stability of adherens junctions and is associated with suppression of tumor progression. Therefore, aberrant changes in the *N*-glycosylation process are associated with the development of various forms of cancer [14].

Previous studies from our lab have highlighted the role of cell surface *N*-glycans and their impact on cellular properties. Parental Pro^−^5 Chinese Hamster Ovary (CHO) cells, and glycosylation mutant Lec1 cells, which predominantly express complex or oligomannose *N*-glycans, respectively, [15,16] were utilized. It was observed that oligomannose and complex *N*-glycans, as well as complex with bisecting *N*-glycans, each had profound effects on the spatial arrangement of E-cadherin in the plasma membrane and E-cadherin dependent cell–cell adhesion [7,8]. Here we have conducted a systematic and simplified approach to directly compare the effects of hybrid, complex, and oligomannose types of *N*-glycans on cellular properties. We employed the clustered regularly interspaced short palindromic repeats/caspase9 (CRISPR/Cas9) technique, a recently established technology [17,18], to knock out *Mgat2* in Pro^−^5 cells. This glycosylation mutant CHO cell line is referred to as the K16 cell line and it is the first established glycosylation mutant cell line that predominantly expresses hybrid type *N*-glycans. Results of this study demonstrate the importance of oligomannose, hybrid, and complex types of *N*-glycans at the cell surface and their effects on cellular architecture and properties, and thus health and disease of mammals.

## 2. Results

**Figure 1:**
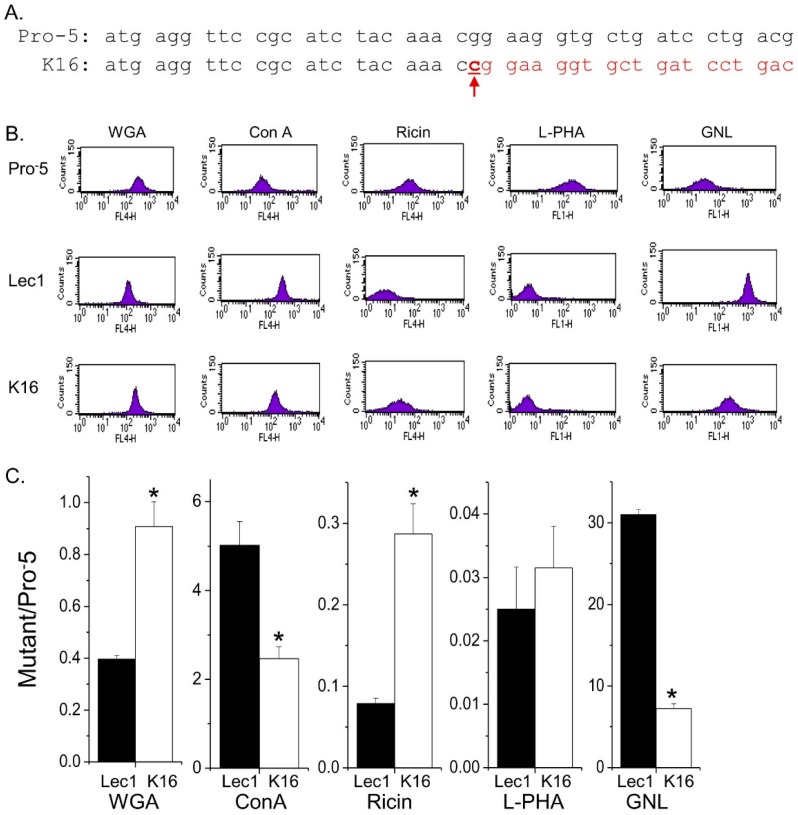
Characterization of an *N*-glycosylation mutant Pro^−^5 Chinese Hamster Ovary (CHO) cell line generated by employment of the CRISPR/Cas9 method. The coding sequence (CDS) of the *Mgat2* gene from 1 to 42 is shown for the parental cell line, Pro^−^5, and the isolated clonal cell line, K16 (**A**); K16 has the Mgat2 gene silenced due to insertion of c after the 22nd nucleotide, as denoted by the red arrow. The red font indicates the changes in the codon as a result of the insertion; Representative flow cytometry histograms of five fluorescently labelled lectins binding to each of the CHO cell lines (**B**); Ratios of mean fluorescence values of the *N*-glycosylation mutant cell lines to the parental cell line were determined and compared from four separate experiments (*n* = 4) (**C**). Mean ratio values close to 1 indicate that the binding of the *N*-glycosylation mutants were similar to Pro^−^5. (*, *p* < 0.02) denote that K16 was significantly different from Lec1.

Current knowledge on the cellular roles of hybrid type *N*-glycans is quite limiting since glycosylation mutant cell lines for the *Mgat2* gene have not been established until now. Here we compare the role of hybrid type *N*-glycans to the other two general types of *N*-glycans (e.g., complex and oligomannose) in controlling cellular properties. The parental, Pro^−^5, and *N*-glycosylation mutant, Lec1, CHO cell lines predominantly express complex and oligomannose types of *N*-glycans, respectively. The mutant cell line that predominantly expresses hybrid type *N*-glycans was engineered using the CRISPR/Cas9 method. This newly created cell line has the *Mgat2* gene silenced, and will be referred to as the K16 cell line throughout the text. The K16 cell line was identified and confirmed by DNA sequencing of nine separate cell clones. The coding sequence of the *Mgat2* gene had a C residue inserted after the 22nd nucleotide residue, resulting in a frameshift mutation (Figure 1A). It should be noted that this mutation introduces nonsense amino acid sequence and numerous early premature stop codons. Also, an alternative start site that would be in frame is not present until amino acid residues 98 of the wild type coding sequence of GlcNacT-II.

To verify that the type of *N*-glycans expressed by the K16 cell line is predominantly hybrid *N*-glycans, lectin binding of this cell line was compared to the Pro^−^5 and Lec1 cell lines. Our selection of cell lines allowed simultaneous characterization of all three types of *N*-glycans. Further cellular changes in the cell lines should be primarily due to the type of *N*-glycans expressed since the two *N*-glycosylation mutants are derived from the same parental cell line. Fluorescently labelled lectins, one at a time, were bound to each of the three cell lines, and the level of fluorescent intensity was determined using flow cytometry. The lectins utilized were wheat germ aggluttin (WGA), concanavalin A (ConA), Ricinus communis agglutinin I, RCA 120 (Ricin), *Phaseolus vulgaris* Leucoagglutinin (L-PHA), and *Galanthus nivalis* Lectin (GNL). The specificity of ConA and GNL are directed towards mannose residues, and furthermore GNL has higher affinity for oligomannose *N*-glycans [16,19]. WGA interacts with GlcNAcβ1-4GlcNAcβ1-4GlcNAc and Neu5Ac structures which could be part of hybrid or complex types of *N*-glycans while L-PHA has preference for complex type of *N*-glycans over the other two types of *N*-glycans [16]. Ricin binds Galβ1-4GlcNAcβ1-R structures, and therefore should have preference for complex type, followed by hybrid type, and least of all oligomannose type *N*-glycans [16]. Representative flow cytometry data is shown for the parental and *N*-glycosylation mutant cell lines with the five different lectins, as indicated (Figure 1B). The flow cytometry plots clearly show that WGA binds to the cell surface of Lec1 cells with much less affinity than both Pro^−^5 and K16. Both ConA and GNL bound at a much higher level to Lec1 and K16 cells than Pro^−^5 cells, and furthermore both of these lectins had the highest affinity for Lec1 cells. The binding of L-PHA to Lec1 and K16 cells was greatly reduced relative to Pro^−^5 cells. Ricin binding to the mutants was also decreased compared to Pro^−^5 cells, although K16 cells had higher affinity for this lectin than Lec1. The bar graph demonstrates that K16 cells have similar affinity for WGA, higher affinity for GNL and ConA, and much reduced affinity for L-PHA and ricin relative to Pro^−^5 cells (Figure 1C). Additionally, the K16 cell line also has a different lectin binding profile than the Lec1 cell line. For instance, both GNL and ConA bind with much higher affinity to Lec1 cells than K16 cells while ricin has higher affinity for K16 cells than Lec1 cells. The binding of L-PHA to both cell lines is quite low.

**Figure 2:**
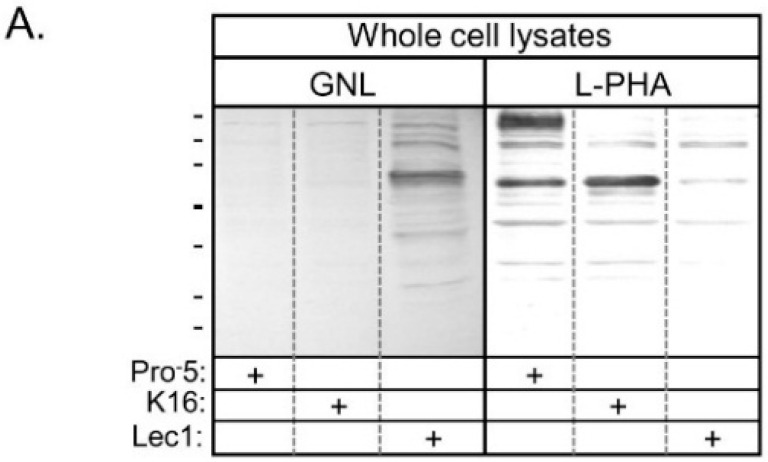

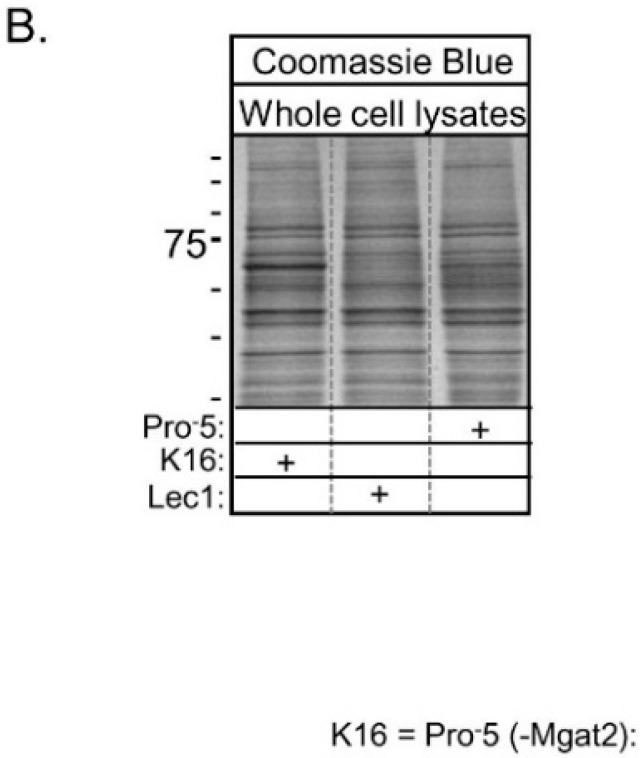
Lectin blots of whole cell lysates from parental and *N*-glycosylation mutant CHO cell lines. Pro^−^5, K16, and Lec1 samples were probed with *Galanthus nivalis* Lectin (GNL) and *Phaseolus vulgaris* Leucoagglutinin (L-PHA), as indicated (**A**); SDS gels with similar levels of protein were evaluated by coomassie blue staining (**B**). Plus signs signify the cell line examined. Lines adjacent to blot and gel denote molecular weight standards in kDa: 250, 150, 100, 75, 50, 37, 25 and 20 from top to bottom.

Lectin blots of whole cell lysates from Pro^−^5, K16, and Lec1 cell lines verified that GNL has highest affinity for glycans of proteins generated in Lec1, intermediate affinity for those in K16, and lowest affinity for those in Pro^−^5 (Figure 2A). Blots using L-PHA revealed highest affinity for glycans of Pro^−^5, and quite low affinity for glycans made by K16 and Lec1 cell lines. A coomassie blue stained gel representative of identical loads as the lectin blots revealed that the levels of protein loaded per well were quite similar (Figure 2B). Based on the lectin binding profiles, the results confirm that Pro^−^5 expresses predominantly complex type *N*-glycans, while the Lec1 cell line expresses oligomannose type *N*-glycans [15,16]. Furthermore, the current results support that the *Mgat2* gene is silenced in the K16 cell line, and therefore that hybrid type *N*-glycans are predominately synthesized in this newly engineered cell line.

### 2.1. Effects of N-Glycan Types on Cell–Cell Adhesion and Cell Migration

**Figure 3:**
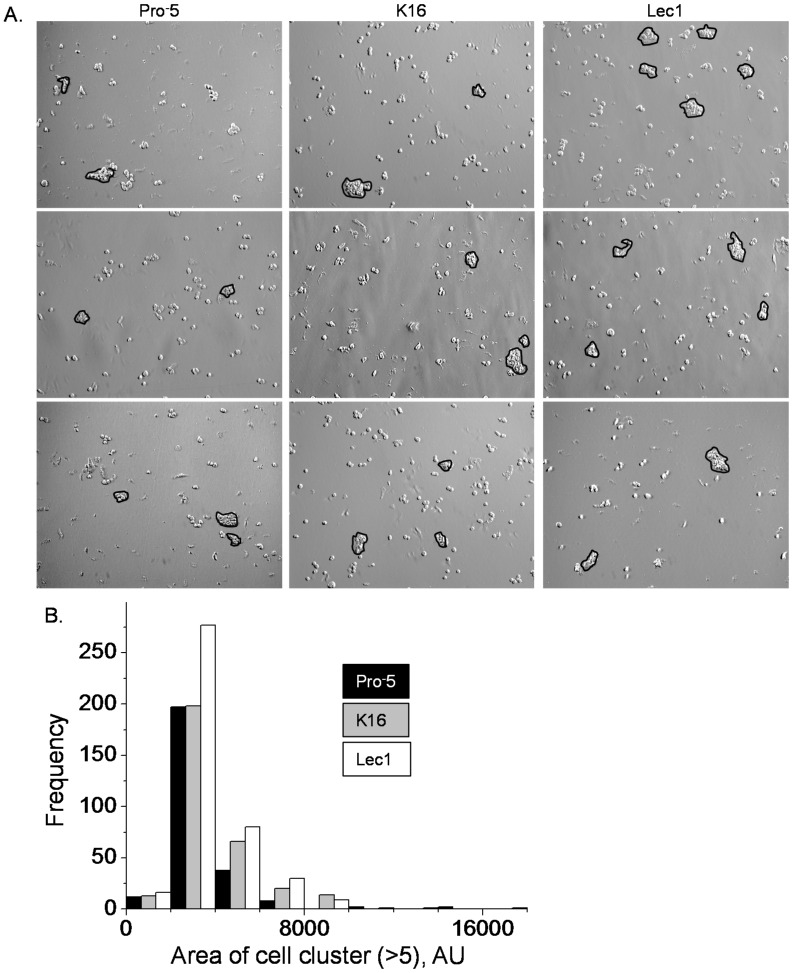
Type of *N*-glycan influences cell–cell adhesion. Microscopy images were acquired for Pro^−^5, K16, and Lec1 cell lines (**A**); Particles (>5 cells/cluster) examined are encircled in black. The histogram represents the area of cell clusters from about 200 images for each cell line, as indicated (**B**), AU, arbitrary unit.

Previous studies have revealed that complex and oligomannose *N*-glycans have profound effects on the development of mammals [3,4,20] and cellular properties [7,8,9,10,21]. Here we directly compared cell–cell adhesion and cell migration properties due to changes in the expression of hybrid type *N*-glycans with those of complex and oligomannose types of *N*-glycans. Cell–cell adhesion was examined by employment of the cell dissociation assay. Representative images are shown for CHO cell lines that predominantly express complex (Pro^−^5), hybrid (K16), or oligomannose (Lec1) types of *N*-glycans (Figure 3A). Dissociation of the Lec1 cell monolayer produced more cell clusters (>5 cells/cluster) than either the K16 or Pro^−^5 cell lines (Figure 3B). The mean number of cell clusters per image were 1.36 ± 1.10 AU (arbitrary unit) (*n* = 198), 1.56 ± 0.09 AU (*n* = 202), and 2.08 ± 0.14 AU (*n* = 199) for Pro^−^5, K16 and Lec1 cell lines, respectively. The occurrence of small cell clusters (≤4000 AU) produced by dissociation of the K16 cell monolayer was similar to that of the Pro^−^5 cell line but less than that of the Lec1 cell line (Figure 3B). On the other hand, the K16 cell line, like the Lec1 cell line, had more large cell clusters (>4000 AU) than the Pro^−^5 cell line. The mean area of cell clusters were 3404 ± 99 AU (*n* = 259), 3906 ± 110 AU (*n* = 316), and 3765 ± 87 AU (*n* = 415) for Pro^−^5, K16, and Lec1 cell lines, respectively. Therefore, hybrid type *N*-glycans impact cell–cell adhesion characteristics differently than those of complex or oligomannose types of *N*-glycans in epithelial-derived cells. Specifically, the strength of cell–cell adhesion is from greatest to weakest in CHO cells expressing oligomannose, hybrid, and complex types of *N*-glycans, respectively.

**Figure 4:**
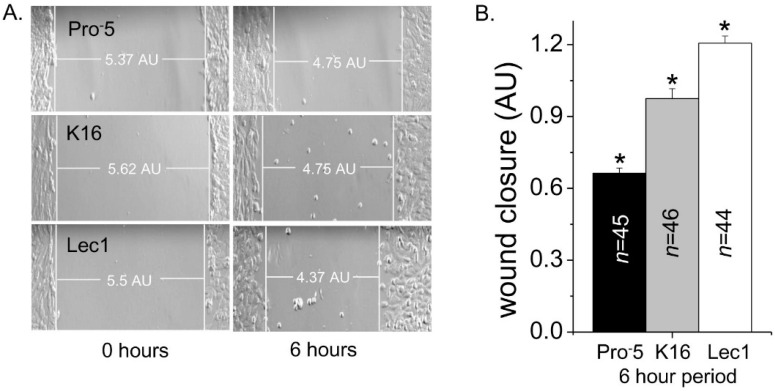
Rate of CHO cell migration is altered by type of *N*-glycan. Images were captured at 0 and 6 h time points for parental and *N*-glycosylated mutant CHO cell lines, as indicated (**A**); rate of cell wound closure was determined for the various CHO cell lines (**B**). White lines at the leading edge of the cell monolayers, and the horizontal line connecting these two vertical lines represent the width of the cell wound. *n* denotes the number of cell wounds measured from four separate experimental days. Asterisks indicate significant differences in mean values at a probability of *p* < 0.03 using one-way ANOVA with Bonferroni adjustments.

Cell migratory rates as a function of *N*-glycan types were monitored by cell wound healing assays. Selected images at 0 and 6 hours are shown for Pro^−^5, K16, and Lec1 cell lines (Figure 4A). It could be observed that cell wounds from cells expressing predominantly complex type *N*-glycans closed the slowest, those with hybrid type were intermediate, and those with oligomannose were fastest. Mean values of the cell wound closures show that the migratory rates for each of the cell lines were significantly different (Figure 4B). Taken together, the results indicate that oligomannose type *N*-glycans enhance the migratory rate of CHO cells to a greater degree than hybrid type *N*-glycans and to a much greater degree than complex type *N*-glycans.

### 2.2. Expression of E-Cadherin with Hybrid Type N-Glycans

**Figure 5:**
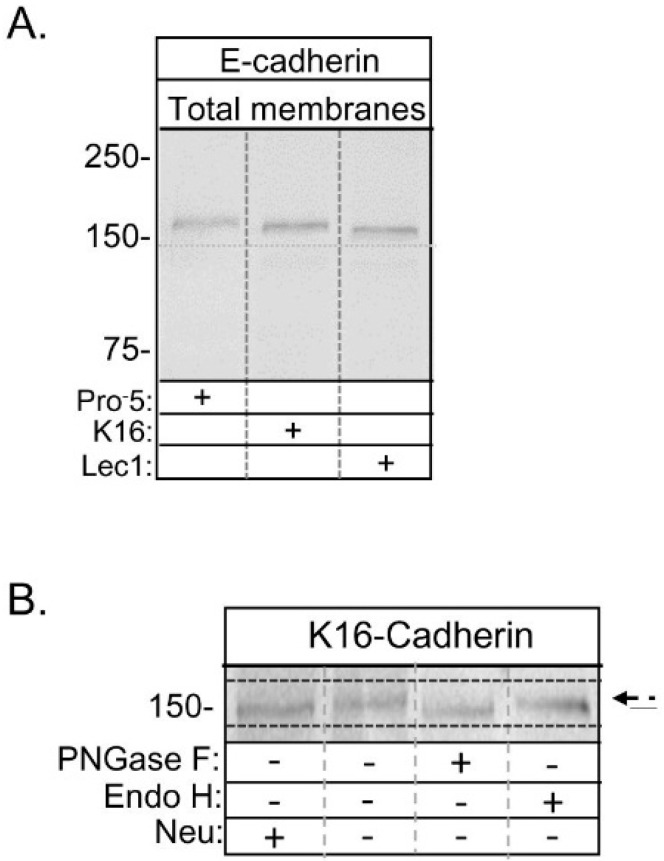
Electrophoretic migration of E-cadherin expressed in the various CHO cell lines. Western blots of E-cadherin glycoprotein in total membranes from transfected Pro^−^5, K16, and Lec1, as denoted by the plus sign (**A**); dashed line on blot was utilized to illustrate small electrophoretic shifts for the three immunobands. Total membranes of E-cadherin transfected K16 cell line were digested (+) and undigested (−) with PNGase F, Endo H, and neuraminidase (neu) (**B**); dashed arrow indicates glycosylated E-cadherin and solid line represents the unglycosylated form. Solid lines below and above immunobands were employed to emphasize the small electrophoretic shifts. The numbers adjacent to the Western blots represent the Kaleidoscope markers (in kDa).

Recently, we showed that the *N*-glycans of heterologously expressed E-cadherin were of complex and oligomannose types in stably transfected Pro^−^5 and Lec1 cell lines, respectively [7]. The type of *N*-glycan correlated with the predominant type of *N*-glycan expressed by each of the cell lines [15,16]. Here we compared the electrophoretic migration of glycosylated E-cadherin heterologously expressed in the newly engineered K16 cell line to those of glycosylated E-cadherin heterologously expressed in the other two cell lines (Figure 5A). The glycosylated form of E-cadherin expressed in K16 cells (≈158 kDa) migrated faster than that expressed in Pro^−^5 cells (≈162 kDa), and slower than that expressed in Lec1 cells (≈154 kDa). Glycosidase digestion reactions of total membranes from E-cadherin transfected K16 cells showed that the electrophoretic migration could be increased for samples treated with PNGase F while increased migration of E-cadherin in samples treated with Endo H was lacking (Figure 5B). This indicated that *N*-glycans were of complex or hybrid types since they could be removed by PNGase F but not Endo H. Further there was a small downward electrophoretic migratory shift due to digestion of the sample with neuraminidase, which could suggest that at least one of the antennae is capped with sialic acid or that E-cadherin is *O*-glycosylated. These data, combined with results of the engineered K16 cell line (Figure 1 and Figure 2), and our previous characterization of the E-cadherin transfected Pro^−^5 and Lec1 cell lines [7], support that the glycosylated form of E-cadherin heterologously expressed in the K16 cell line has a hybrid type *N*-glycan. As such, the *N*-glycans of E-cadherin will be referred to as complex, hybrid, and oligomannose types in Pro^−^5, K16, and Lec1 cell lines, respectively, throughout the text.

### 2.3. Impact of Distinct N-Glycan Types on Sub-Plasma Membrane Localization of Functional E-Cadherin

**Figure 6:**
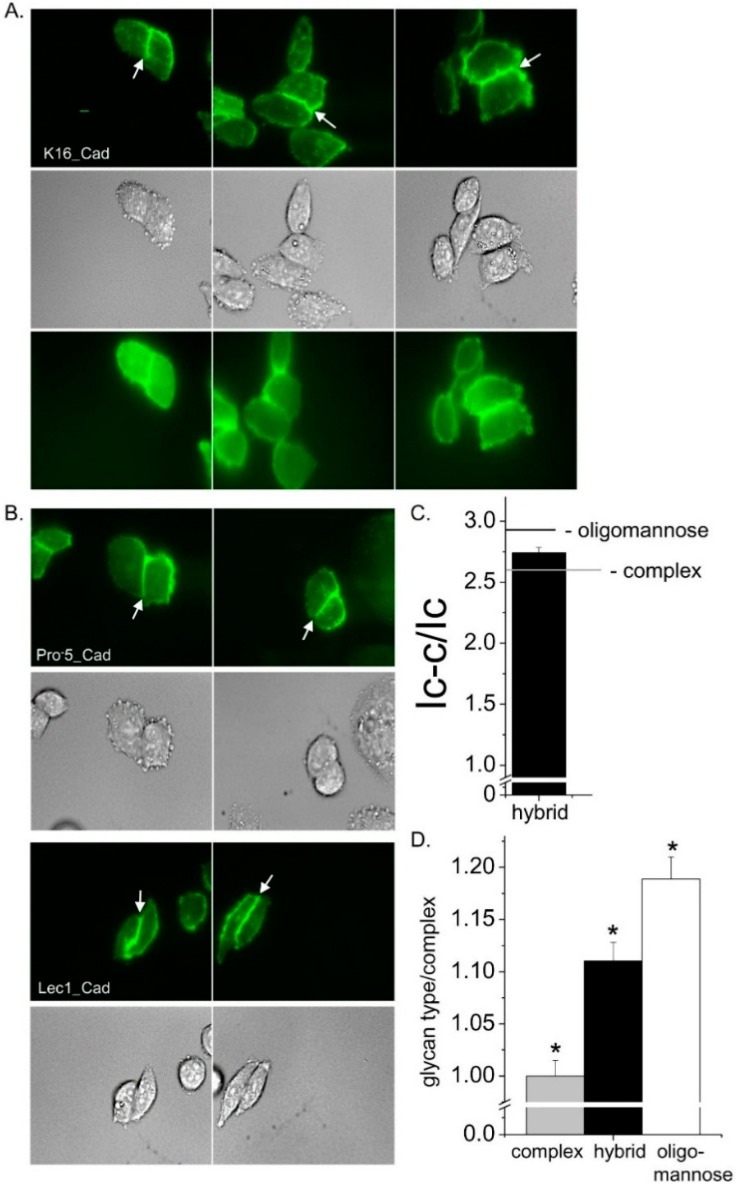
Predominant expression of hybrid *N*-glycans impacts the localization of E-cadherin at the cell–cell border in a different manner than predominantly expressed complex or oligomannose *N*-glycans. Microscopy images were acquired in TIRF (**upper panels**), wide-field (**middle panes**), and DIC (**lower panels**) modes for EGFP tagged E-cadherin expressed in K16 cell line (**A**). To directly compare the localization of E-cadherin to the cell–cell interface with complex and oligomannose *N*-glycans, TIRF (**upper panels**) and DIC (**lower panels**) images were also acquired for E-cadherin transfected Pro^−^5 and Lec1 cell lines (**B**); Representative scale bar (5 µM) was same for all images. Cell–cell interface is denoted by white arrows. Fluorescence intensity measurements were ascertained at the cell–cell interface (I_cell–cell_), and away from the cell–cell interface (I_cell_) of the cell membrane patch to determine the amount of E-cadherin at cell–cell border relative to that away from the cell–cell border (I_cell–cell_/I_cell_). The bar on the graph reports the I_cell–cell_/I_cell_ mean value of E-cadherin expressed in the K16 cell line while the lines designated as complex and oligomannose are the mean values for E-cadherin expressed in Pro^−^5 and Lec1 cell lines, respectively (**C**); The ratio of I_cell–cell_/I_cell_ value for a given *N*-glycan type to complex type of *N*-glycan illustrates differences in the spatial arrangement of E-cadherin expressed with different predominant *N*-glycan types (**D**). At the *p* < 0.01 level, the differences of the population means are significantly different by one-way ANOVA with Bonferroni adjustment (*).

High contrast images of live K16 cells expressing glycosylated E-cadherin tagged with enhanced green fluorescent protein (EGFP) at the plasma membrane were acquired by employment of total internal reflection fluorescence (TIRF) microscopy (Figure 6A, top panel). The fluorescent signal appeared to accumulate to the cell–cell border. Further the placement of the intense fluorescent signal at the cell–cell border is supported by accompanied differential interference contrast (DIC) images (Figure 6A, middle panel). The wide-field images have a much more diffuse signal than the TIRF images, and also the signal was less intense at the cell–cell border relative to that away from the cell–cell border (Figure 6A, bottom panel). These results substantiate that the fluorescent signal in the TIRF mode is from EGFP tagged E-cadherin in or near the plasma membrane. Previously, we showed that more E-cadherin glycoprotein was at the cell–cell border when the *N*-glycans were of oligomannose type than complex type [7]. As a reference and for a direct comparison, TIRF and DIC images of E-cadherin heterologously expressed in the Pro^−^5 and Lec1 cell lines were also acquired using similar parameters (Figure 6B). Both cell lines expressed E-cadherin predominantly at the cell–cell border; however more E-cadherin was at this border when oligomannose was the predominant *N*-glycan as previously described [7]. The level of E-cadherin at the cell–cell border relative to that away from the cell–cell border (I_cell–cell_/I_cell_) for E-cadherin transfected K16 cells was in between the two control cell lines (Figure 6C). The significance of these differences is illustrated by taking the ratios of the I_cell–cell_/I_cell_ values for each type of the glycans to the complex type (Figure 6D). Therefore, these results indicate that the three types of *N*-glycans modulate the level of E-cadherin at the cell–cell border relative to that away from the cell–cell border.

**Figure 7:**
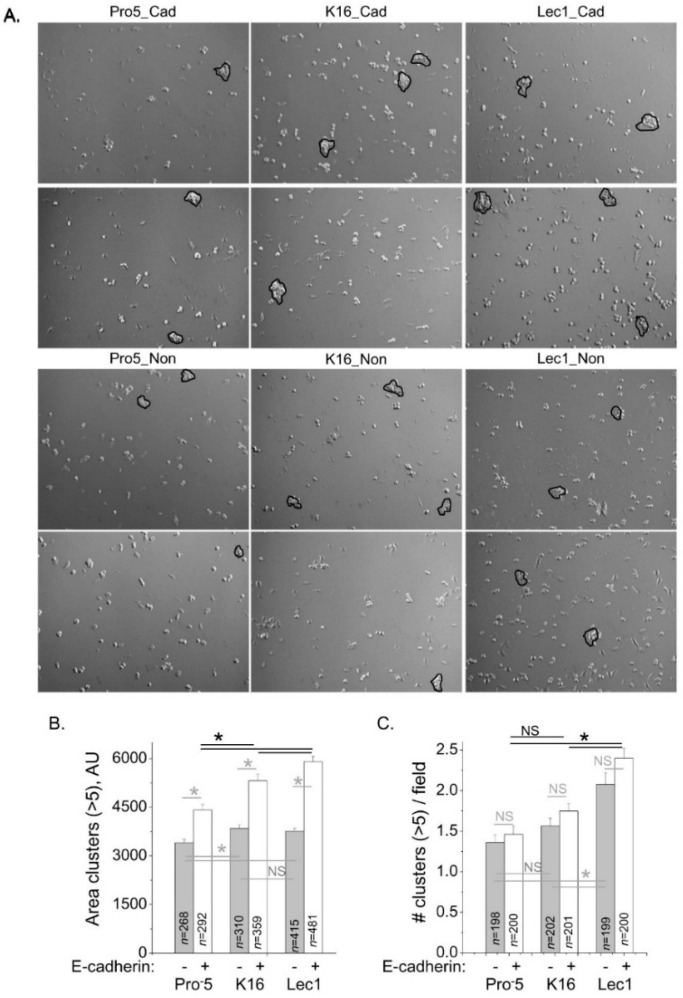
E-cadherin modified cell–cell adhesion in cells expressing different types of *N*-glycans. Microscopy images were acquired for E-cadherin transfected Pro^−^5 and non-transfected Pro^−^5, K16, and Lec1 (**A**); particles (>5 cells per cell cluster) analyzed are encircled. Mean values of the area (**B**), and number (**C**) of cell clusters were plotted on bar graphs from four separate days of experiments, as indicated. *n* values were greater than 199 fields and 267 clusters. Black asterisks denote significant differences in mean values of a non-transfected cell line, and E-cadherin transfected cell line at a probability of *p* < 0.00001 using student *t*-test (**B**). Gray asterisks represent significant differences in mean values of non-transfected or E-cadherin transfected CHO cell lines at a probability of *p* < 0.05 using one-way ANOVA with Bonferroni adjustments. NS signifies mean values were not significantly different.

To correlate E-cadherin at the cell–cell border with functional E-cadherin, we evaluated cell–cell adhesion of the K16 cell lines heterologously expressing E-cadherin, along with the two control cell lines. Representative images from cell dissociation assays are shown for E-cadherin transfected Pro^−^5 (left panels), K16 (middle panels) and Lec1 (right panels) cells (upper two panels), and same cell lines that were not transfected with E-cadherin (lower two panels) as controls (Figure 7A). For each field, we analyzed cell clusters with more than five cells. The mean values of area of clusters were significantly different between CHO cell lines expressing E-cadherin (Figure 7B). Further the area of clusters increased with the increased expression of E-cadherin at the cell–cell border. It was observed that heterologous expression of E-cadherin in all three cell lines increased the size of the clusters relative to the absence of E-cadherin expression (Figure 7B). The mean value of number of clusters for K16 cells was not significantly different to that of Pro^−^5 cells but lower than that of Lec1 cells whether E-cadherin was expressed or not expressed (Figure 7C). Taken together, these results support that hybrid type *N*-glycans have more E-cadherin at the cell–cell border than complex type *N*-glycans, and less at the cell–cell border relative to oligomannose type *N*-glycans, and furthermore that the level of E-cadherin correlates with amount of functional E-cadherin.

### 2.4. Influence of E-Cadherin on Cell Migratory Rates

**Figure 8:**
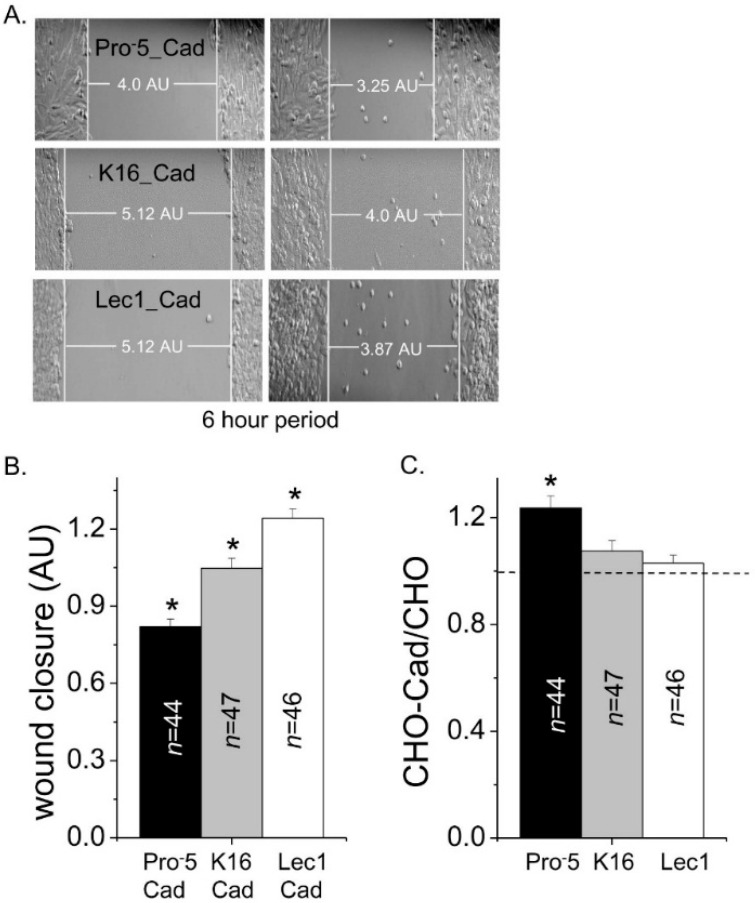
Influence of CHO cell migration by E-cadherin. Images were captured at 0 and 6 h time points for parental and *N*-glycosylated mutant CHO cell lines heterologously expressing E-cadherin, as indicated (**A**); rate of cell wound closure was determined for the various transfected CHO cell lines (**B**); cellular migratory rates in cell lines expressing E-cadherin were normalized to their respective non-transfected cell line (**C**). White lines at the leading edge of the cell monolayers, and the horizontal line connecting these two vertical lines represent the measured width of the cell wound. Asterisks indicate significant differences in mean values at a probability of *p* < 0.03 using one-way ANOVA with Bonferroni adjustments. Asterisk above line denotes significant differences in mean values at a probability of *p* < 0.03 using student *t*-test.

Since the glycosylated form of E-cadherin modified cell–cell adhesion, it was determined whether these different glycosylated forms altered cell migration. Representative images from cell migration assays are shown for E-cadherin transfected Pro^−^5 (upper panels), K16 (middle panels), and Lec1 (lower panels) cell lines (Figure 8A). Cell wounds for the transfected K16 cell line closed slower than the transfected Lec1 cell line and faster than the transfected Pro^−^5 cell line (Figure 8B). Heterologous expression of E-cadherin in Pro^−^5 cells enhanced cell migratory rates compared to the non-transfected Pro^−^5 cells while the presence of E-cadherin in K16 or Lec1 cell lines did not influence their cell migratory rates (Figure 8C). These results indicate that the role of a given glycosylated form of E-cadherin on cell migratory rates is influenced by the predominant type of *N*-glycan expressed by the CHO cell line.

## 3. Discussion

The role of carbohydrate–carbohydrate interactions have been implicated in cell–cell adhesion and cell migration. In the current study, the function of hybrid type *N*-glycans was compared to the function of complex and oligomannose types on cell adhesion and motility. CHO cell models were used since the glycans in the parental Pro^−^5 and glycosylated mutant Lec1 CHO cell lines have been well-characterized [15,16]. The aforementioned studies revealed that Lec1 solely expressed oligomannose type *N*-glycans, and the predominant type of *N*-glycan expressed by Pro^−^5 was complex. Here the CRISPR/Cas9 technology [17,18] was employed to engineer a glycosylation mutant K16 CHO cell line that predominantly expresses hybrid type glycans. Up to now, from our knowledge, a cell line that predominantly expresses hybrid type *N*-glycans was unavailable. We observed that oligomannose type *N*-glycans have a greater contribution to the adhesion of cells to each other, and faster migratory rates than hybrid type *N*-glycans while complex type *N*-glycans had the least contribution on these two cellular properties. Thus, our study underlines the cellular roles of the various types of *N*-glycans in the development of mammals as described in mice with either *Mgat1* [3] or *Mgat2* [4] knocked out.

Carbohydrate-dependent interactions that modulate cell adhesion, motility, and signaling have been described as three distinct types of glycosynapses [5,6]. The type 3 glycosynapse (e.g., integrin-tetraspanin-ganglioside complex) involves N-linked glycans of an integrin receptor while glycosphingolipids and *O*-linked glycans of mucin type glycoproteins are the major contributors of the type 1 and 2 glycosynapses, respectively [5,6]. Our current study focuses on disruptions of either *Mgat1* or *Mgat2*, which solely altered the type of *N*-glycans, not *O*-glycan structures. Verification that *Mgat* genes only alter *N*-glycans is supported by *N*-glycan and *O*-glycan profiles in various CHO cell lines [15]. The major cell adhesion molecule expressed by CHO cells is the α5β1 integrin [22], and GM3 (monosialodihexosylganglioside) is the major glycolipid [23,24]. As such, our results indicate that changes in the types of *N*-glycans may directly alter cell migration via the type 3 glycosynapse. Further it may be that different forms of complex type *N*-glycans impact the type 3 glycosynapse since increased branching of complex type *N*-glycans enhanced α5β1 integrin-mediated cell migration [25,26]. It is also plausible that the cellular roles of the other two glycosynapses are indirectly altered by redistribution of these components in the plasma membrane.

Previous studies have indicated that carbohydrate–carbohydrate interactions provide strength and specificity for cell–cell adhesion [27]. E-cadherin significantly dominates cell–cell adhesion, thereby adding to the difficulty in assessing the contribution of cell surface *N*-glycans to this process. Consequently, our current study employed various CHO cell lines that do not endogenously express E-cadherin [28], and also they do have similar *O*-glycan profiles [15]. Therefore, our results provide evidence that changes in complex type of *N*-glycans at the cell surface to hybrid or oligomannose types enhance the strength of cell–cell adhesion with the later having the greatest strength. This enhancement could be due to direct carbohydrate–carbohydrate interactions of the *N*-glycans, and/or changes in the arrangement of cell surface glycans, including glycolipids, in the plasma membrane.

Distinct *N*-glycan structures guide the placement of *N*-glycosylated transmembrane proteins in the plasma membrane in a protein-specific manner [7,8,9,10,21,29]. Herein, we found that processing of the *N*-glycans from oligomannose type to hybrid type decreased the level of E-cadherin at the cell–cell border and the amount of E-cadherin at this border was further decreased when the *N*-glycan was processed to a complex type. Further when a bisecting N-acetylglucosamine was added to the complex *N*-glycan, the level of E-cadherin was lowest [7]. Previously, endogenously expressed galectin-1 [30] was shown to interact with glycoconjugates at the cell surface [31] of CHO cells. Since galectins participate in cell-matrix and cell–cell interactions [32], it may be that galectin-1, or perhaps other galectins, are contributing to the spatial arrangement of E-cadherin in the adhered plasma membrane. This is also supported by binding studies of galectins, including galectin-1, with various CHO cell lines, showing that decreased branching of *N*-glycan structures results in reduced galectin–glycan interactions [33]. Thus, we propose that enhanced interactions of galectins with complex type *N*-glycans may localize more E-cadherin away from the cell–cell border. Further, our proposal supports a role of galectins in mediating cellular interactions with extracellular matrix since we examined the spatial arrangement of E-cadherin in the adhered plasma membrane. However, it should be noted that the interactions of galectins with different types of *N*-glycans appear to be dependent on protein structure in localizing *N*-glycosylated transmembrane proteins to or away from the cell–cell border since the spatial arrangement of Kv3.1b, a voltage-gated potassium channel, in the adhered plasma membrane of the various parental and glycosylated mutant CHO cell lines was different than those for E-cadherin [7].

Previously, we showed that higher accumulation of E-cadherin with oligomannose *N*-glycans at the cell–cell border correlated with enhanced cell–cell adhesion while E-cadherin with complex type *N*-glycans had less functional E-cadherin at the cell–cell border [7]. The aforementioned studies suggested that the conversion of *N*-glycans from oligomannose type to complex type reduced E-cadherin-mediated cell–cell adhesion. Herein, it was shown that the addition of one branch site (action of GlcNAcT-I) to the conserved pentasaccharide reduces the level of functional E-cadherin at the cell–cell border, and that the addition of a second branch point (action of GlcNAcT-II) further reduces the strength of E-cadherin-mediated cell–cell adhesion. Our study on the impact of all three general types of *N*-glycans in E-cadherin-mediated cell–cell adhesion can be extrapolated to the role of E-cadherin in adherens junctions since E-cadherin with higher levels of oligomannose *N*-glycans supports the establishment of stable adhesion junctions while higher levels of complex *N*-glycans drastically reduced these junctions [12].

## 4. Materials and Methods

### 4.1. Generation of CHO Cell Line with Mgat2 Silenced

The CRISPR/Cas9 technology was employed to silence *Mgat2* in the Pro^−^5 cell line as described previously [17,18]. In brief, the sgRNA oligonucleotides (5′-CACCGTTCCGCATCTACAAACGGA-3′ and 5′-AAACTCCGTTTGTAGATGCGGAAC-3′) were selected using the Zi-Fit Targeter software [34,35]. After phosphorylation and annealing of oligonucleotide, double-stranded gRNA molecules were cloned into the pSpCas9(BB)-2A-Puro vector (Addgene plasmid ID: 48139), and sequencing confirmed. The expression vector was transfected into Pro^−^5 cells using lipofectamine 2000 per the manufacturer’s instructions or as we previously described [36]. After transfection, cells were treated with 4 µg/mL puromycin for 48 h. Clonal cell lines were selected and *Mgat2* silencing was confirmed by DNA sequencing of targeted genomic region.

### 4.2. Cell Culture and Transfection

Parental Pro^−^5 and glycosylation mutant Pro^−^Lec1 (Lec1) CHO cells were purchased from American Type Culture Collection (Manassas, VA, USA). Stably transfected E-cadherin K16 CHO cell line was generated as previously described for E-cad Pro^−^5 and Lec1 [7]. CHO cells of 60%–70% confluency were transfected with neomycin selectable expression plasmids encoding E-cadherin for generation of stable cell lines as previously described [7]. Cells were cultured in MEM Alpha Media (Hyclone, Logan, UT, USA) with 10% fetal bovine serum, 50 µg/mL streptomycin and 50 U/mL penicillin (Gemini BioProducts, West Sacramento, CA, USA) under 5% CO_2_ at 37 °C.

### 4.3. Lectin Binding Analysis by Flow Cytometry

CHO cells were incubated with 10 µg/mL of either fluorescein tagged lectin (Vector Laboratories, Inc., Burlingame, CA, USA) Phaseolus vulgaris leucoagglutinin (L-PHA), or Galanthus nivalis lectin (GNL); or rhodamine tagged lectin wheat germ agglutinin (WGA), concanavalin A (ConA) or Ricinus communis agglutinin I, RCA 120 (Ricin) for 15 min at room temperature. Fluorescence intensity was acquired using a FACS Vantage flow cytometer (Becton Dickinson Biosciences, San Jose, CA, USA) using 488 nm laser excitation and emission centered at 530 nm for fluorescein tagged lectins and 560 nm laser excitation and emission centered at 575 nm for rhodamine tagged lectins. Mean fluorescence values were determined from histogram plots of fluorescence emission and results were normalized relative to the parental (Pro^−^5) cell line.

### 4.4. Glycosidase Digestions

Total membranes were isolated from CHO cells as previously described [7]. In brief, cells (≈1.35 × 10^8^) were suspended in lysis buffer (10 mM Tris, pH 7.4; 250 mM sucrose, 5 mM EDTA; protease inhibitor cocktail set III (Calbiochem, San Diego, CA, USA) 1:500), homogenized, centrifuged, and then supernatant was collected and subsequently centrifuged at 100,000× *g* for 1 h. Pellet was resuspended in lysis buffer and protein concentration was determined by Lowry assay. Glycosidase digestions of total CHO membranes (5 g/L) were incubated with 0.83 U/µL neuraminidase, 20 U/µL PNGase F, and 50 U/µL Endo H in supplied buffers (New England Biolabs, Ipswich, MA, USA). Reactions were incubated at 37 °C for about 16 h and then reaction halted by adding reducing SDS-PAGE sample buffer.

### 4.5. Western and Lectin Blots

E-cadherin total membrane samples for western blotting and whole cell lysates for lectin blotting were run for 1.7 h on 10% SDS gels. Proteins were transferred to PVDF membranes (Millipore, Billercia, MA, USA) for 3.5 h at 100 V or 4 h at 250 mA. Incubations and development of blots were as described [7]. Rabbit pan Cadherin antibody (Novus Biologicals, Littleton, CO, USA) was employed to detect E-cadherin.

### 4.6. TIRF Microscopy

Cells were plated onto 35 mm poly-l-lysine coated glass bottom dishes (MatTek, Ashland, MA, USA) and incubated for 25–26 h. Total internal reflection fluorescence (TIRF), differential interference contrast (DIC), and wide-field images of the cells were obtained with an ORCA R2 deep cooled mono CCD camera attached to an Olympus IX 71 microscope (Olympus, Center Valley, PA, USA) equipped with a Apo 60× 1.45 objective as described [7]. An argon laser beam of wavelength 488 nm was used to excite live cells Images were captured with an ORCA R2 deep cooled mono CCD camera through an Apo 60× 1.45 objective. Exposure time of 1000 ms was used. CellˆTIRF Control 1.1 and Metamorph for Olympus Basic software was used to control shutters, filters, and camera. Image J software was employed for data analysis.

### 4.7. Dissociation Assays

Cells were plated at similar concentrations and grown for 2 days on 35 mm CellBind culture dishes (Corning, Corning, NY, USA) [7]. At confluency, cells were washed twice and then resuspended in media. Cells were detached with a cell scraper, and then cell aggregates were dissociated by pipetting fifteen times. Images (30–35 fields/dish) were acquired on an Olympus IX 71 microscope with a 10× objective. Particles (≥5 cells/aggregate) were counted, and their areas determined by employment of Image J software. Data is given as the mean ± S.E. and n denotes the number of particles. Statistical comparison was evaluated via student’s *t*-test. Statistical significance was considered at *p* < 0.05.

### 4.8. Wound Healing Assays

Cell migration experiments were conducted as previously described [7]. Cells were plated in equal concentrations and allowed to grow to confluence, at which time the media was aspirated and wounds were created in the cell monolayer by employment of a beveled 200 µL pipet tip. Cells were washed twice with media and images were obtained at 0 and 6 h on an Olympus IX 71 microscope using a 10× objective. The average wound closure (AU) due to the predominant *N*-glycan of each cell line was ascertained by taking the difference in wound closure between the initial width and final width of the wound. The average wound closure due to E-Cadherin was determined by taking the difference in wound closure between the E-cadherin transfected and non-transfected, and dividing the difference by the closure of the non-transfected.

## 5. Conclusions

We conclude that hybrid type *N*-glycans, like complex and oligomannose type *N*-glycans, contribute to cellular properties in a glycan specific manner. Additionally, each glycan type impacts membrane architecture by guiding the unique placement of E-cadherin in the plasma membrane. Consequently, future studies to further explore how changes in *N*-glycan structures impact the maintenance of an organism and in disease progression will be of great benefit.
